# Public awareness and attitudes towards epilepsy in Tehran, Iran

**DOI:** 10.3402/gha.v6i0.21618

**Published:** 2013-12-05

**Authors:** Helia Ghanean, Marzieh Nojomi, Lars Jacobsson

**Affiliations:** 1 Department of Psychiatry, Umea University, Umeå, Sweden; 2 Department of Public Health, Tehran Medical University, Tehran, Iran

**Keywords:** epilepsy, attitudes, knowledge, stigma, Iran

## Abstract

**Background:**

Epilepsy is a prototypical, stigmatised disorder. Numerous studies have been conducted regarding the public perception of epilepsy, but they are primarily from high-income western countries; few studies have taken place in low- to middle-income countries with a traditional culture and a religious orientation.

**Objective:**

The public knowledge and attitudes towards epilepsy in Tehran, Iran, is studied.

**Design:**

A survey of 800 subjects ranging from 18 to 85 years was randomly chosen from households in Tehran in 2009. The questionnaire used was based on the Caveness and Gallup's studies conducted in the United States in 1949 and it has been used in numerous similar studies all over the world. The mean age of the participants was 37.5 years and 46.7% were female. Pearson's Chi-squared test was used for subgroup analyses.

**Results:**

The majority of subjects cited brain disorders as a cause of epilepsy, while 17% indicated the will of God as the cause. Most individuals were willing to work with a person with epilepsy, allow their children to play with a child with epilepsy, and allow people with epilepsy to use public transportation (78–82%). However, only 28% were willing to accept the marriage of a family member to someone with epilepsy.

**Conclusion:**

The knowledge and attitudes towards epilepsy are similar to those in Europe, with the exception of a much lower acceptance regarding marriage to a person with epilepsy. However, the low acceptance for marrying someone with epilepsy reveals the remaining misconceptions about the nature of epilepsy in Iran, despite the high educational level in the studied population. Therefore, informational efforts must be employed to change the perception of epilepsy.

‘The history of epilepsy can be summarised as a 4000 years of ignorance, superstition and stigma, followed by 100 years of knowledge, superstition and stigma’ (1). Epilepsy is a prototypical, stigmatised disorder. Uncontrolled seizures can be very dramatic and have serious consequences for both the afflicted person and for those close to him/her. For example, falling into a fire due to an epileptic fit can cause serious injuries and result in the destruction of the home of a person suffering from epilepsy. The disorder is described in very early history and is usually connected with demons, spiritual possession, and other supernatural forces. A study by Vanzan and Paladin on epilepsy and Persian culture refers to Avesta, a collection of Zoroastrian texts from the 6th century B.C. that refers to a sickness called epilepsy. In this document, God tells Zoroaster that people suffering from epilepsy are prohibited from offering sacrifices (2).

People suffering from epilepsy have been discriminated against in several ways (3, 4). In numerous countries, families try to hide the disorder to allow the person with epilepsy and other family members to marry (5, 6). People with epilepsy find it difficult to obtain jobs. Procuring a driver's license is often problematic. People with epilepsy often fail to obtain health insurance in many countries.

In high-income countries with modern treatment facilities and a more educated public, the problems caused by the stigmatisation of epilepsy have decreased (7). In low-income countries, the ‘treatment gap’ deters many people suffering from epilepsy from obtaining proper treatment; consequently, the disorder remains a major, public health problem. The World Health Organisation has launched a global campaign against epilepsy (GCAE) called ‘Out of the shadows’ (8). Two main methods have been suggested to reduce the stigma of epilepsy: improved treatment and changes in the public perception of the disorder using information campaigns.

The first studies on the public awareness and attitudes towards epilepsy were performed in the United States in 1949 by Caveness and Gallup (9, 10). They developed a set of questions that has since been used in several follow-up studies in the United States and in many other studies worldwide (11). These studies have shown that public perception has changed, albeit slowly (7). Most of these studies were performed in high-income western countries. However, several studies have indicated that the stigma for epilepsy is similar in low-income countries and traditional cultures where the causes of epilepsy are still often considered to be evil forces, spiritual possession, broken taboos, and other devaluating conditions (7, 12, 13). An interesting possibility of a lower level of stigma would be in Muslim cultures, where the Koran clearly states that people suffering from different types of disorders should be treated with respect because their fate might be attributed to the will of Allah rather than personal weaknesses or sinful behaviour. According to Vanzan and Paladin, the Koran and Mohammed's sayings ‘do not contain any explicit references to epilepsy’; other texts by Mohammed's followers have no mention of epilepsy as a sickness caused by demons (2).

A number of studies have been conducted in Islamic countries. A study by Bener et al. from the United Arab Emirates showed a rather high awareness of epilepsy, but 40% believed that faith healing is the proper treatment. A small percentage (7%) objected to allowing their children to play with children with epilepsy, but up to 68% objected to their children marrying a person suffering from epilepsy (14). The same response pattern was found in a study from Iran (5). The attitude towards children marrying a person suffering from epilepsy was negative (54–75%). However, Iranians had a positive attitude about employing people suffering from epilepsy and their association with healthy people. These data are rather interesting; in general, the attitudes are accepting, but not in the case of marriage.

People suffering from epilepsy were historically considered to have a changed personality; they were considered to be unstable, explosive, hypochondriacs, and clingy (15). A common way to talk about people suffering from epilepsy is to refer to them as ‘epileptic,’ meaning that epilepsy sufferers are reduced to being their disorder rather than a person with a disorder. Furthermore, epilepsy is only a symptom; several neurological disorders and disorders that change brain activity cause epileptic fits.

This study investigated the public knowledge and attitudes towards epilepsy in Tehran, Iran.

## Methods

The greater Tehran area contains nearly 12 million inhabitants (16). Tehran is divided into 22 districts and 112 sub-districts (nahiyeh) that are divided into blocks (howzeh). A total of 800 literate individuals over the age of 15 years were recruited using a multistage sampling method in randomly chosen districts from the northern, eastern, southern, and western areas of Tehran. A sample containing 600–800 people was considered necessary (Jackknife method) (17).

A questionnaire was delivered by four trained psychologists from the university – two male and two female. One person per household was asked to fill out the questionnaire. The investigators waited while the questionnaire was completed to offer help if there were any questions. The investigators approached the families on weekday mornings and afternoons, as well as on holidays, to obtain representative samples. The data collection occurred from April to December in 2009.

The questionnaire addresses socio-demographic background data, age, education, marital status, and occupation, and includes 14 questions regarding the knowledge and attitude towards people suffering from epilepsy and one open-ended question regarding the cause of epilepsy. This set of questions has been used in several studies worldwide, and it is based on the original study by Caveness in the United States (9).

The questionnaire was translated into Farsi from the English version through translation and back-translation by psychiatrists and neurologists knowledgeable in English.

The project was approved by the ethics committee of the Medical School of the Tehran University of Medical Sciences. The study was conducted in accordance with the Helsinki declaration on research ethics. Participation was voluntary, and the responses were anonymous.

## Data analysis

The data were analysed using the SPSS v 13 software. The frequency distribution was calculated, and Pearson's Chi-squared test was used for subgroup analysis. Variables were considered significant at the 0.05 level.

## Results

The socio-demographic background data for the population are presented in Table 1. The mean age of the population was 37.5 years and ranged from 15 to 85 years. There were slightly more males (53%) than females. The females were often married and stayed at home to care for the household.

**Table 1:** Socio-demographic background of the population (%)

		Males (*n*=426)	Females (*n*=374)	p*
Age	≤25 years	24.4	24.7	
	26–45 years	38.7	38.2	
	46–60 years	29.1	34.0	
	>60 years	7.7	3.5	<0.05
Education**	< Diploma (<12)	23.0	23.8	
	Diploma (12)	38.5	35.6	
	>Diploma (>12)	38.5	40.6	n.s.
Marriage	Single	40.4	30.7	
	Ever married	59.7	69.2	<0.01
Occupation	Occupied	74.4	31.8	
	Non-occupied/house wife	25.6	68.2	<0.001

* Chi-square with 95% CI.

** Years at school.

The subjects’ knowledge about epilepsy is presented in Table 2. Generally, the majority had observed an epileptic fit (74%) and knew someone with epilepsy (58%). A few connected epilepsy with insanity (9%).

**Table 2:** Knowledge about epilepsy – yes responses (*N*=800)

Have you ever heard of epilepsy?	76.6%
Do you know of any other word for epilepsy in your language?	61.0%
Have you ever observed an epileptic fit?	74.1%
Do you have a relative who suffers from epilepsy?	23.9%
Do you know anyone with epilepsy?	58.1%
Do you think epilepsy is a form of insanity?	9.4%

One question was about words used for epilepsy; in Farsi, Arabic, and the Turkish language, epilepsy is called ‘Saraa,’ meaning ‘being knocked down.’ Other mentioned words included ‘Taashanog,’ which means ‘moving attack,’ and ‘Ghash,’ which means ‘suddenly falling (knocked) down.’

Regarding the causes of epilepsy (Table 3), the majority mentioned brain diseases (62.2%), while 53% cited two or more alternatives, indicating that they have a rather informed view regarding epilepsy. Seventeen percent named the will of God as a possible cause, and none cited evil spirits.

**Table 3:** Causes of epilepsy (*N*=800) (%)

Infection	3.7
Mental illness	16.9
Accident/head trauma	5.6
Will of God	16.9
Evil spirits	0
Genetic	47.1
Alcohol	0
Brain disease	62.2

The attitudes towards epilepsy are presented in Table 4 and seem to be rather positive. The subjects were willing to work with a person with epilepsy, allow their children to play with a child with epilepsy and allow people with epilepsy to use public transportation (82–78%). However, with regard to accepting someone with epilepsy as a family member through marriage, only 28% agreed.

**Table 4:** Attitudes towards epilepsy (percent yes responses) (*N*=800)

If you had the opportunity, would you employ someone with epilepsy?	67.4
Are you willing to work with a person with epilepsy?	80.0
Would you allow your child to play with child with epilepsy?	82.5
Would you be afraid of someone with epilepsy?	11.4
Would you allow anyone in your family to marry someone with epilepsy?	28.0
Should your family hide a family member with epilepsy from the outside world?	10.3
Do you think a person with epilepsy should use public transportation?	78.1

There were significant gender differences regarding the willingness to employ or work with someone with epilepsy and to allow their child to play with a child with epilepsy. The women were more willing to allow their child to play with a child with epilepsy (87.2 vs. 78.4% for men).

Subjects with more education were generally more accepting towards employing someone with epilepsy, allowing their child to play with a child with epilepsy and allowing a person with epilepsy to use public transportation. However, when accepting someone in the family to marry somebody with epilepsy, there was no significant difference regarding gender or educational level. Interestingly, the oldest group (>65 years) was significantly more accepting than the youngest group (43.5 vs. 25.8%). Conversely, the elderly group was the most likely to hide the fact that someone has epilepsy.

## Discussion

The general knowledge about epilepsy seems to be good in this sample, and the views regarding the causes of epilepsy indicate a fairly knowledgeable population. The vast majority cited a brain disease as a cause of epilepsy (62.2%). More than half of the population provided two or more alternatives, indicating that the subjects have a differentiated view on the possible background. Moreover, 17% cited the will of God, and none blamed evil spirits. This result contrasts with that found in Muslim countries in Africa and other parts of Asia where the idea of evil forces is prevalent (6, 18–20).

Tables 5 and 6 list other studies from different parts of the world that used the same questions about attitudes and knowledge. Regarding the attitudes towards epilepsy, as well as the general knowledge about epilepsy, the Tehran population is similar to populations from high-income countries, such as Austria (11). However, with regard to the question about someone in the family marrying a person with epilepsy, the figures are low, similar to the Masoudnias study about the attitudes and awareness of different Iranian ethnic groups (54–75%) (5). However, even in Austria, only 48% agreed to this possibility. Interestingly, there were no differences in response to this question related to educational level, highlighting the emphasis on family status in Iran.

**Table 5:** Attitudes towards epilepsy in other countries (percent ‘yes’ responses)

Attitude towards epilepsy	Taiwan 1995*	Austria 2005**	Ethiopia 2000***	Tehran 2009
If you had the opportunity, would you employ someone with epilepsy?	35	84	24	67
Would you allow your child to play with a child with epilepsy?	57	82	60	83
Would you allow someone in your family to marry someone who has epilepsy?	13	48		28
Are you willing to work with a person with epilepsy?	35	84	35	80

* Chung et al. 1995 (19)

** Spatt et al. 2005 (11)

*** Shibre et al. 2008 (20).

**Table 6:** Knowledge about epilepsy in other countries (percent ‘yes’ responses)

Knowledge	Hong Kong 2002*	Austria 2005**	Ethiopia 2008**	Tehran 2009
Have you ever heard of epilepsy	58	89	70	77
Do you know of any other word for epilepsy in your language?	19	40	52	61
Have you ever observed an epileptic fit?	55	36	52	74
Do you think epilepsy is a form of insanity?	10	11	41	9

* Fong & Hung 2002 (21)

** Spatt et al. 2005 (11)

*** Shibre et al. 2008 (20).

This last result is interesting and is shared with many populations all over the world. What causes this hesitation to include a person with epilepsy into a family? In many countries, even in the west, there were legal prohibitions against marriage until the middle of the 20th century. One reason may be that people think that epilepsy is hereditary; this prohibition was intended to prevent the spread of the bad ‘genes’ to new individuals. Another reason might be that people with epilepsy are strongly stigmatised, making it shameful to include such a person in a family. This attitude also reflects the importance of family and the effort to protect the family from unwanted, marginalised elements, particularly in the traditional, collectivistic culture found in places such as the Islamic Republic of Iran.

When studying the experience of stigma by people with epilepsy in Tehran, Iran, many individuals experienced stigma and discrimination because they suffered from epilepsy (22). Specifically, 56% agreed that ‘people discriminate against me because I have epilepsy,’ 43% stated that ‘negative stereotypes about epilepsy keep me isolated from the “normal” world’ and 28% agreed that ‘having epilepsy has spoiled my life.’ Therefore, the attitudes towards people with epilepsy in Iran must be changed. Whether Islamic teaching reduces the stigma of epilepsy remains to be studied.

In general, different approaches have been suggested to combat stigma and discrimination against epilepsy (1, 8, 13). One method is to support people with epilepsy by countering negative stereotypes through their member associations and allowing them to argue for better services and less discrimination. Increasing knowledge in the general population regarding the nature of epilepsy and possible treatments might also help. The third and probably most important strategy is to improve the treatment and care of people suffering from epilepsy by providing the appropriate medical treatment and monitoring the disorder, which could decrease the number of epileptic fits and reduce the most apparent signs of the disorder.

## Strengths and limitations

The studied population is representative of the general population in Tehran, including the rapidly growing and well-educated middle class in Iran. The questions used in this study have been used all over the world for many years to study the public awareness and attitudes towards epilepsy, making these a well-established tool. The small differences observed in the proportion of males and females are not particularly important in the results. The majority of the females are unemployed/housewives, reflecting the socio-cultural milieu in Iran.

## Conclusion

The knowledge and attitudes towards epilepsy are similar to those found in studies conducted in high-income western countries, with the exception of the lower acceptance of a person with epilepsy marrying into the family. Therefore, the perception of epilepsy must be changed in Iran. Although people seem to be well informed about the nature of epilepsy, informational campaigns might help change the perception of epilepsy because modern treatment has considerably decreased its medical and social consequences. Possible actors in this would be the Iran Epilepsy Association in collaboration with the Ministry of Health, professional organisations, and media profiting from the experiences of the WHO campaign against stigma because of epilepsy.
